# Periodontal condition and recurrence of periodontitis associated with alcohol consumption in periodontal maintenance therapy

**DOI:** 10.4317/jced.56166

**Published:** 2020-02-01

**Authors:** Fernando-Oliveira Costa, José-Roberto Cortelli, Adriana-Moreira Costa, Rafael-Paschoal-Esteves Lima, Sheila-Cavalca Corteli, Otávio-Miranda Cota

**Affiliations:** 1 Department of Dental Clinics, Oral Pathology, and Oral Surgery, School of Dentistry, Federal University of Minas Gerais, Belo Horizonte, Minas Gerais, Brazil; 2 School of Dentistry, Department of Periodontology, Federal University of Minas Gerais, Brazil; 3 Newton Paiva Institute, Faculty of Dentistry, Belo Horizonte, Minas Gerais, Brazil

## Abstract

**Background:**

This study followed individuals in periodontal maintenance therapy (PMT) over 6 years and longitudinally evaluated the effects of the frequency of alcohol consumption on the recurrence of periodontitis (RP).

**Material and Methods:**

From a 6-year follow-up cohort study with 268 individuals under PMT, 142 patients who attended at least one PMT visit within 12 months were determined to be eligible. Based on their alcohol consumption, participants were categorized into 3 groups: none or occasional alcohol use (NA; n=88), moderate alcohol use (MA; n=26) and intense alcohol use (IA; n=24). Complete periodontal examination and alcohol consumption were evaluated at 2 times, T1 (after active periodontal therapy) and T2 (6 years).

**Results:**

The frequencies of RP in the NA, MA and IA groups were 46.5%, 57.6%, and 79.1%, respectively. The following variables were significantly associated with RP in final multivariate logistic regression model: age >50 years old (OR = 1.79; 95%CI 1.42-2.91; p=0.002), current smoking (OR = 2.42; 95%CI 1.33-4.31; *p*=0.001), and intensive alcohol use (OR = 1.96; 95%CI: 1.37-2.64; *p*=0.024). Interaction between intensive alcohol use and smoking showed a high OR estimate of 3.15 (95%CI 1.29-6.32) for RP.

**Conclusions:**

IA individuals undergoing PMT presented worse periodontal condition, higher rates of RP and tooth loss when compared to NA individuals. Additionally, the interaction between intensive alcohol use and smoking significantly increased the risk for RP.

** Key words:**Periodontitis, alcohol consumption, maintenance, epidemiology.

## Introduction

Periodontitis is a non-communicable disease characterized by microbially-associated, host mediated inﬂammation resulting in loss of periodontal attachment and can lead to tooth loss (TL) (1). Periodontal disease onset and propagation happens through the dysbiosis of the commensal oral microbiota (dental plaque), which then interacts with the immune host defenses, leading to inflammation and disease (2). The severity of periodontitis depends on environmental and host risk factors, both modifiable (i.e. smoking, alcohol consumption, obesity, life style factors, social life status and unhealthy conditions) and non-modifiable (i.e. age and genetic susceptibility) (2).

Alcohol consumption as a risk factor has been studied in relation to a great variety of conditions since it is responsible for almost 4% of deaths worldwide and almost 5% of the global disease contingent (3). It is also an important cause of health inequalities among individuals under negative social impact, due to high cost and specific care demand (4). A great number of diseases and injuries are directly caused by alcohol consumption (4,5).

In recent decades, observational and epidemiological studies (5-9) pointing to a potential association between alcohol consumption and the development and progression of periodontitis have increased. A recent systematic review (10) reported that studies evaluating this relation are numerous and comprise different designs, yet presenting conflicting data. Furthermore, the authors emphasized that more well-designed cohort studies are necessary to conﬁrm this risk association.

Several studies have highlighted the benefits of periodontal maintenance therapy (PMT) to preserve the homeostasis of periodontal tissues obtained after active periodontal therapy (APT), performed through surgical and/or non-surgical procedures (11-14) However, without establishing a regular program of clinical re-evaluation, adequate biofilm control, and re-inforcement of oral hygiene instructions, the benefits of PMT could not be maintained and a higher risk for future recurrence of periodontitis (RP) (12-14) and TL may surge (14,15). Besides, no prospective PMT study has demonstrated the effect of the frequency of alcohol consumption on periodontal condition and RP.

The hypothesis under testing in the present study was that individuals with frequent alcohol consumption higher RP, TL and worse periodontal clinical condition during PMT.

Therefore, the present study followed individuals in PMT for over 6 years and longitudinally evaluated the effects of the frequency of alcohol consumption on periodontal condition and RP.

## Material and Methods

-Study Design and Sampling Strategy

Participants of the present prospective study were selected from an open cohort study with 268 individuals under a PMT program, who were monitored in a private dental clinic in the city of Belo Horizonte – Brazil, over 6 years of consecutive recall visits (from August 2006 to February 2016). The study was approved by the local ethical committee (protocol #060/05) and a written informed consent was obtained from all participants. This study was reported in accordance with the STROBE statements guidelines.

Individuals that underwent APT (comprised of non-surgical and/or surgical procedures) were included in the study sample according to the following criteria: (a) diagnosis of moderate to advanced chronic periodontitis (16,17) (excluding any possibility of aggressive periodontitis cases) – prior to APT, the presence of least 4 sites with probing depth (PD) ≥5mm and clinical attachment loss (CAL) ≥3mm, bleeding on probing (BOP) and/or suppuration (SU), and radiographic evidence of bone loss; (b) completion of APT in a period of less than 4 months prior to entering the PMT program; and (c) at least 14 teeth in the oral cavity (12). It is noteworthy that, based on the new classification system of periodontal diseases, an update in periodontal status of the sample was performed and individuals were currently classified with moderate to severe periodontitis (1).

From the cohort study with 268 individuals under PMT, 138 individuals who attended at least one recall visit within 12 months during the study period and completed the questionnaires of alcohol consumption between T1 (data being recorded after the first PMT appointment) and T2 (final data being recorded at the last PMT appointment, e.g., after 6 years under PMT) were determined to be eligible, thus representing a convenience sample.

These individuals were then stratified according to the frequency of alcohol consumption by means of 2 questionnaires: CAGE (Cut down, Annoyed, Guilty, Eye-opener) proposed by Mayfield *et al.* (18) and validated by Masur and Monteiro (19) and AUDIT (Alcohol Use Disorders Identification Test) proposed by the World Health Organization) (20).

The AUDIT questionnaire (20) composes itself by 10 questions, having scores >8 indicating alcohol use problems. The CAGE questionnaire (18,19) is made of four questions, having scores ≥2 indicating alcohol dependence. In this manner, individuals were categorized according to AUDIT and CAGE scores in three groups: (1) none or occasional alcohol use – never used or frequency of use less than monthly, AUDIT and CAGE scores = 0; (2) moderate alcohol use – frequency of use 2–4 times a month, AUDIT score ≤8 and CAGE score = 0; (3) intense alcohol use – frequency of use ≥3 times a week, AUDIT score ≥8 and CAGE score ≥1.

Therefore, participants were allocated into: NA group – none or ocasional alcohol use (n = 88); MA group – moderate alcohol use (n = 26); and IA group – intense alcohol use (n = 24).

In order to verify the power of the sample in each group, a sample size calculation was performed considering PD changes (>4mm) as the primary outcome for the recurrence of periodontitis (RP). Considering a significance level of 5%, a study power of 80%, a medium size effect (0.50) and a 15% minimum difference between groups in relation to PD changes (mean values), a calculated number of at least 23 individuals per group was determined to be necessary.

-Data Collection

Baseline data was recorded after the first PMT appointment (T1) and the final data at the last PMT appointment, i.e., after 6 years under PMT (T2).

Parameters of plaque index (PI), PD, CAL, and BOP were recorded for all present teeth at 4 periodontal sites (mesial, distal, buccal, and lingual) with a manual periodontal probe (Hu-Friedy®, Chicago, USA).

Description of data collection and periodontal clinical procedures during all PMT visits were previously reported by Lorentz *et al.* (12) and Costa *et al.* (13).

**Figure 1:**
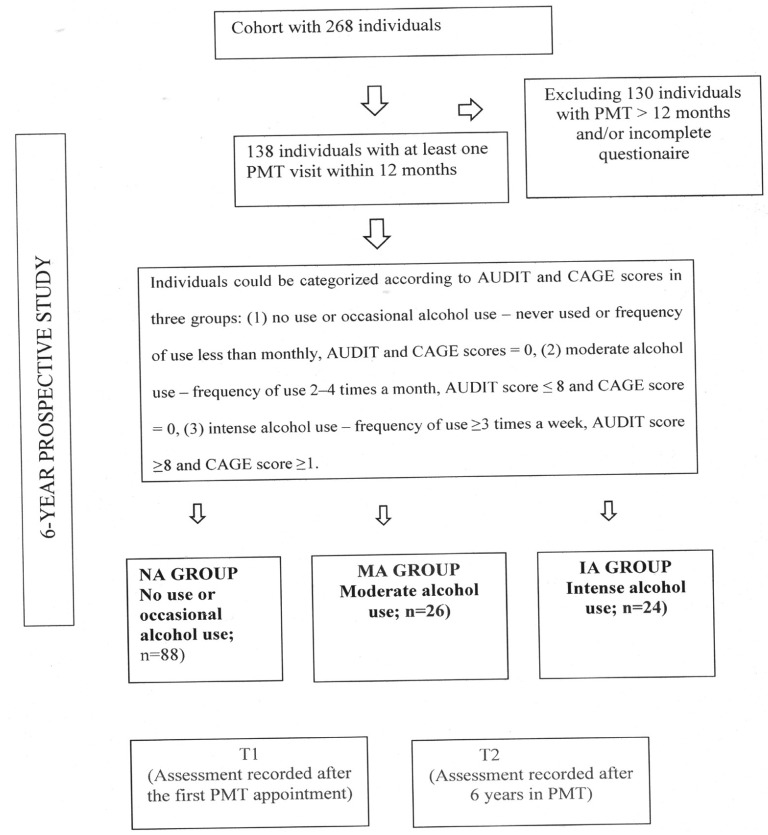
Flowchart of sampling strategy and study evaluation times.

These following variables were also collected: sex, age, family income, co-habitation status, educational level, smoking (29) and diabetes. The flowchart of sampling strategy and study evaluation times is displayed in Figure 1.

-Periodontal monitoring 

In each recall visit, the following procedures were performed: 1) interviews where demographic, biological and behavioral variables of interest were collected and confirmed through patient questionnaires, paying particular attention to those variables likely to change over time; 2) periodontal assessment through the evaluation of clinical parameters described in this paper; 3) application of disclosing agents and oral hygiene instructions, using the Bass technique and dental flossing or adjunctive methods (interdental brushing or water flossing; 4) mechanical debridement, when appropriate, including coronal prophylaxis and fluoride application. All procedures were performed by a group of trained and calibrated professionals.

-Determination of the recurrence of periodontitis (RP) and retreatment needs

Sites determined as having retreatment needs were the ones with RP: PD >4mm and CAL ≥3mm, together with the persistence and/or presence of BOP and/or SU, during any of the subsequent recall evaluations (13,22). PD changes were first re-treated with non-surgical procedures through mechanical subgingival debridement. After periodontal re-evaluation (45 to 60 days), sites with persistent PD ≥5mm and CAL ≥3mm underwent surgical procedures using the Widman modified flap technique (13).

-Inter- and intra-examiner agreement

Two trained and calibrated periodontists (FOC and EJPL) performed all the interviews, examinations, and clinical periodontal procedures. Evaluations of PD and CAL were performed and repeated within a 1-week interval in 10 individuals randomly selected from study groups at baseline and at T2. The kappa coefficients for intra- and inter-examiner agreement as well as intra-class correlation coefficients were greater than 0.87.

-Statistical analysis

Statistical analysis included a descriptive characterization of the sample according to variables of interest. Group comparisons by means of the Chi-squared and the Student t tests were performed when appropriate. Multiple comparisons were performed by ANOVA and Welch test and adjusted by the Bonferroni correction post-hoc test.

A logistic regression analysis was performed to evaluate the association between RP and the independent predictor variables. All predictors presenting a *p*-value of 0.25 in the univariate analysis were included in the multivariate regression model. Variables were then manually removed step by step until the log-likelihood ratio test indicated that no variable should be removed. Variables were determined to be confounders if their removal from the model made changes greater than 15% in the B coefficient. Odds ratio (OR) estimates and their 95% confidence interval (CI) were calculated and reported. The quality of the model was determined by measures of sensitivity, specificity, area under the ROC (Receiver Operating Characteristic) curve, Pseudo R² (Nagelkerke) and the Hosmer-Lemeshow test. All tests were performed using statistical software (Statistical Package for Social Sciences, 16.0 – SPSS Inc., Chicago, IL, USA.) Results were considered significant if a *p-value* lower than 5% was attained (*p*<0.05).

## Results

This study comprised a sample of 138 individuals under PMT over 6 years, 24 individuals under intensive alcohol use, 26 individuals under moderate alcohol use and 88 individuals who did not or occasionally used alcohol.

Group characteristics regarding variables of interest are presented in  Table 1. Significant differences between groups were observed in relation to sex, co-habitation status, diabetes, and smoking. However, important variables such as time since APT and number of PMT visits were not significant among the study groups.

**Table 1:**
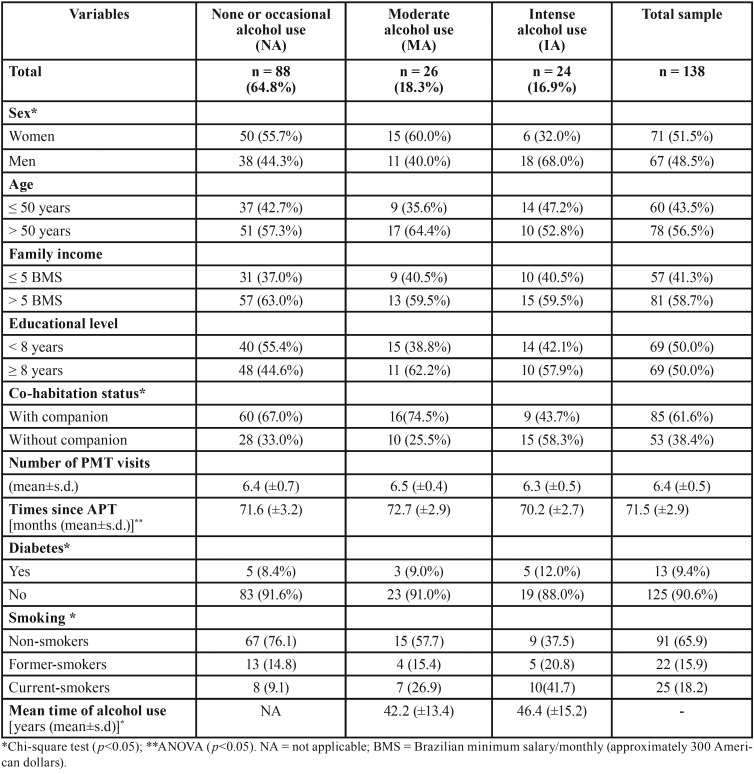
Characteristics of participants by alcohol consumption status at T2.

Comparative analysis among study groups and times in relation to periodontal clinical parameters is presented in  Table 2. Overall, no significant differences were observed regarding the parameters PI, BOP, PD, and CAL among groups at the baseline (IA =MA =NA). As a result, groups were determined to be homogeneous after APT. On the other hand, significant differences between NA, MA, and IA groups were observed at the final examination. The IA group exhibited higher mean PI, BOP, PD, and CAL, as well as higher TL (IA > MA > NA). Moreover, at T2, group IA showed significant differences in relation to group MA for all clinical parameters. However, the comparison of MA versus NA did not reveal significant differences for PI and BOP.

**Table 2:**
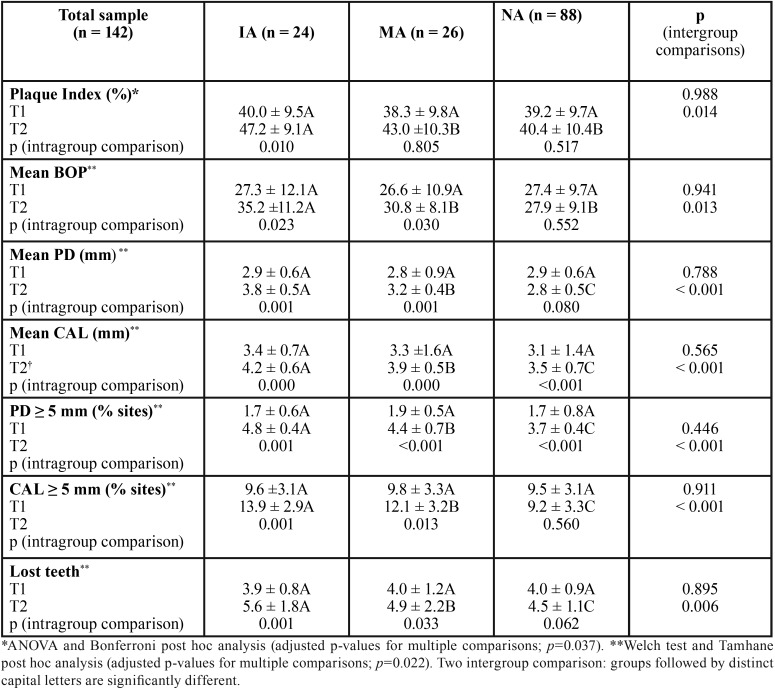
Comparative analysis among study groups and examination times in relation to periodontal clinical parameters.

The frequency of RP was 46.5% (reference) in the NA, 57.6% (crude OR = 1.23; 95%CI 0.83-1.84; *p* = 0.222) in the MA and 79.1% (crude OR = 1.69, 95%CI 1.25-2.30, *p* = 0.04) in the IA group.

In the univariate analysis, age >50 years old (OR = 1.63; 95%CI 1.15-2.31; *p* = 0.002), former smoking (OR = 1.55; 95%CI 1.07-2.24; *p* = 0.020) and current smokers (OR = 1.82; 95%CI 1.34-2.46; *p* = 0.001) were significantly associated with RP ( Table 3).

**Table 3:**
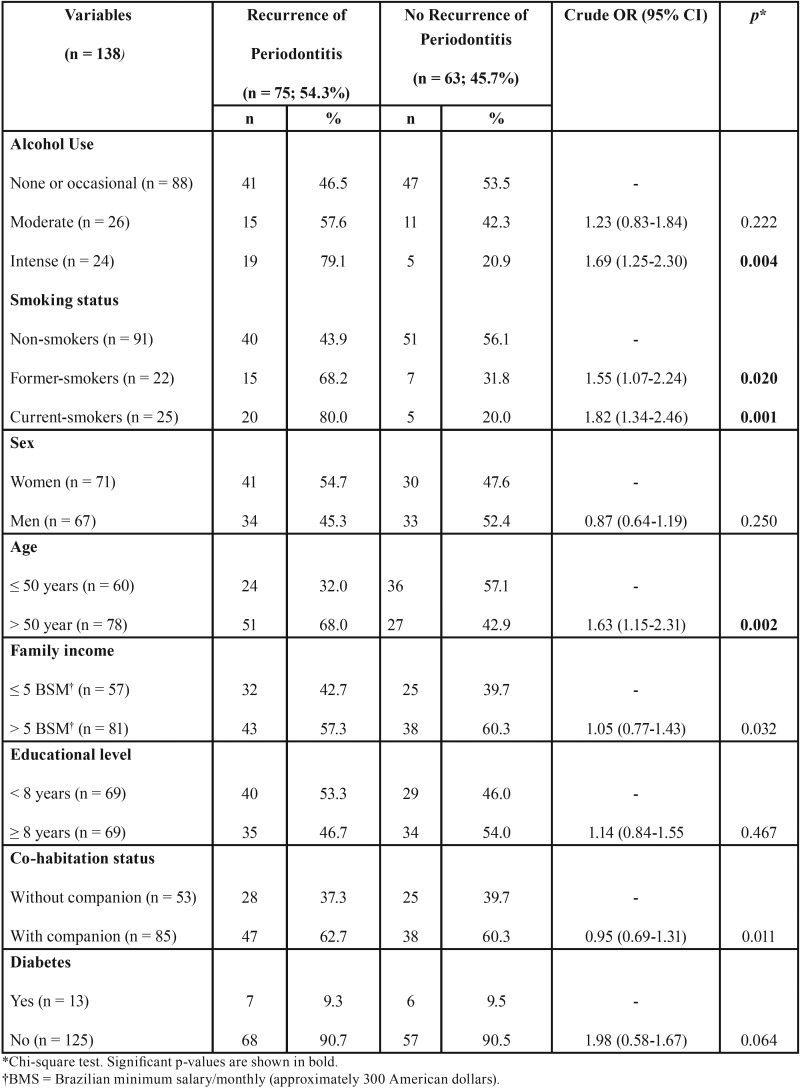
Distribution of independent variables according to the recurrence of periodontitis at T2.

Final multivariate logistic regression model for RP, adjusted for all variables of interest, are shown in  Table 4. The following variables were significantly associated with RP at T2: age >50 years old (OR = 1.79; 95%CI 1.42-2.91; *p*=0.002), current smoking (OR = 2.42; 95%CI 1.33-4.31; *p*=0.001), and intensive alcohol use (OR = 1.96; 95%CI: 1.37-2.64; *p*=0.024). Interaction between intensive alcohol use and smoking showed higher OR estimate of 3.15 (95%CI 1.29-6.32; *p*=0.012) for RP. It is stressed that smokers with intensive alcohol use showed 3 times more chance of presenting PR.

**Table 4:**
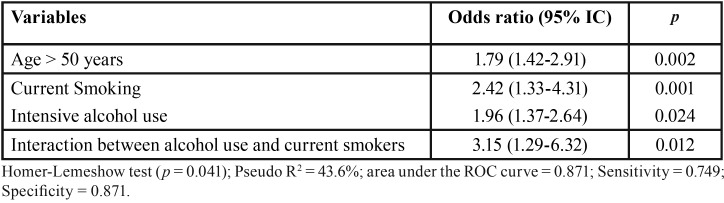
Final logistic regression model for the recurrence of periodontitis at T2.

## Discussion

The present study investigated the association between the frequency of alcohol consumption and RP in individuals on PMT and confirmed the hypothesis of higher alcohol consumption being associated with worse periodontal condition and higher RP and TL. Significant differences between NA, MA, and IA groups were observed at T2. The IA group exhibited higher mean PI, BOP, PD, and CAL, as well as higher TL (IA > MA > NA). Moreover, MA and NA were significantly different only in relation to PI and BOP. It is noteworthy that these findings were more pronounced in smokers.

Conflicting findings regarding the association between alcohol consumption and periodontitis were previously published in the literature, having some studies reporting it as positive (5-7,9,10) while others reporting no association (23,24). Few prospective studies (5,8,25) have evaluated the influence of alcohol on the progression of periodontitis. To our knowledge, no study investigated the effect of alcohol consumption among individuals under PMT.

The mechanisms underlying the association between alcohol consumption and the risk for periodontitis are still unclear. According to the review and meta-analysis from Wang *et al.* (10), the following biological plausibility explanations are listed: “(i) periodontal disease is associated with impaired neutrophil phagocytosis. Thus, alcohol could weaken neutrophil function, leading to bacterial overgrowth and increasing bacterial penetration, which could result in periodontal inflammation; (ii) alcohol intake could result in toxic effects on periodontium and may make a reduction in monocyte production of inflammatory cytokines which are possibly beneficial to microbial proliferation; (iii) the inflammatory cytokines such as TNFa, IL-1 and IL-6 released by monocyte in gingival crevice have been proved to be interrelated in periodontitis development.”

Based on our previous PMT studies (12,13), we believe that recall visits at short intervals may compromise adherence to maintenance programs over the years for different reasons. In the present study, we established a range of recall interval times being the one that the majority of our cohort participants followed during PMT without further worsening the periodontal clinical condition. This interval time (up to 12 months) was yet considered reasonable in clinical practice. Rosén *et al.* (26) suggested that recall intervals extended to a year might be acceptable for the purpose of reducing periodontal disease progression in individuals with a history of limited or moderate susceptibility to the disease.

The present study showed that, in addition to the intensive alcohol use, other variables were also significantly retained in the final multivariate model for RP: age >50 years old, current smoking and the interaction between intensive alcohol use and current smoking.

Age may be a risk indicator for periodontal disease in some populations. However, aging may be related to increased attachment loss and not to periodontitis (13).

Recent systematic reviews (27,28) have demonstrated strong association between smoking and the risk of periodontal attachment loss, as well as bone and tooth loss. In addition, studies have shown incremental OR estimates for the occurrence of periodontitis when the smoking dose-exposure was evaluated (4,21). Studies have reported a high prevalence of smokers among alcohol users (4,7,28,29). Findings from the present study confirms this statement showing a high prevalence of smokers and a strong association between smoking and periodontitis. It was also demonstrated in the present study that the association between periodontitis and alcohol use was independent from smoking. Similar ﬁndings were also reported (4,7).

It is important to highlight that methodological issues may have signiﬁcantly inﬂuenced the conﬂicting results of the association between alcohol and periodontitis previously presented in the literature (4,5). Some studies presented small samples and great variability in the deﬁnition of alcohol dose-exposure, including different cut-off points for the amount and frequency of alcohol consumption (23,24). Others presented less robust deﬁnitions of periodontitis (5,6). Methods of measurement of alcohol exposure levels and dependence are complex and prone to information biases (4,20). However, the use of AUDIT and CAGE questionnaires can minimize these biases. AUDIT was developed under the supervision of WHO (20) and it is composed of 10 questions (three questions about the consumption of alcoholic beverages, four questions of its dependency, and three questions about problems related to its consumption. CAGE is validated in Brazil (19) and it is composed by four questions. CAGE presents a speciﬁcity of 83% and a sensitivity of 88% when the cut-off point of two positive answers was adopted to deﬁne alcoholic beverages.

Limitations can be attributed to the present study as the measurements of alcohol consumption is subject to bias information, as well as the small sample of individuals with RP in the final multivariate analysis. However, the 6-year follow-up period, the prospective design, the use of a structured questionnaire, the adjustment for confounding variables (mainly age), the standardization of the procedures for periodontal treatment and maintenance therapy may minimize the impact of these limitations.

IA individuals undergoing PMT presented worse periodontal condition, high rates of RP and TL when compared to NA individuals. Additionally, the interaction between intensive alcohol use and smoking significantly increased the risk for RP. This finding demonstrated the influence of intensive alcohol use during PMT in maintaining a good periodontal status.
